# Surgical Treatment of Intraductal Papillary Neoplasm of the Bile Duct: A Report of Two Cases and Review of the Literature

**DOI:** 10.3389/fonc.2022.916457

**Published:** 2022-06-23

**Authors:** Binjie Li, Zhiqiang Liu, Zhuo Meng, Mingyang Li, Weijun Tian, Quanyan Liu

**Affiliations:** Department of Hepatobiliary Surgery, Tianjin Medical University General Hospital, Tianjin, China

**Keywords:** surgical treatment, intraductal papillary neoplasm of the bile duct, mutation, clinical features, prognosis

## Abstract

Intraductal papillary neoplasm of the bile duct (IPNB) is a rare bile duct tumor characterized by intraductal papillary or villous neoplasms covered by neoplastic epithelium with fine fibrovascular stalks in the dilated bile ducts (1). Its true etiology remains unknown. Herein, we report two cases of IPNB that underwent surgical resection. The first case was a 66-year-old male who complained of upper abdominal pain for three years. We found obstruction of the common bile duct and dilation of the intrahepatic and extrahepatic bile ducts after MRCP. Laparoscopic hepatic segmentectomy (S2, S3, S4), resection of the common bile duct, cholecystectomy, and hepaticojejunostomy were performed. The second case was a 67-year-old male with asymptomatic dilation of the intrahepatic duct. The patient underwent robot-assisted laparoscopic hepatic segmentectomy (S5, S6, S7, S8), resection of the common bile duct, hepaticojejunostomy and cholecystectomy.

## Background

According to the 2019 World Health Organization (WHO) classification of digestive system tumors, intraductal papillary neoplasm of the bile duct (IPNB) is a rare tumor in the bile duct that is characterized by intraductal papillary or villous neoplasms covered by neoplastic epithelium with fine fibrovascular stalks in the dilated bile ducts (1, 2). IPNB is mainly reported in Far Eastern countries where hepatolithiasis and clonorchiasis are endemic (1). Based on a multicenter analysis, malignant features of tumors are observed in 43% of IPNB cases, and patient prognoses for malignant lesions are worse than for noninvasive lesions (3). Therefore, early identification and resection of lesions are significant, even in asymptomatic patients.

Here, we report two cases of surgical treatment of IPNB with a review of relevant literature; perhaps they could provide some information to understand this rare disease.

## Case Presentation

### Case 1

A 66-year-old man was referred to our hospital due to abdominal pain. Three years ago, he had presented with right upper quadrant abdominal pain, which worsened after meals, and the pain was more frequent in recent months. He had no noteworthy medical or family history. Physical examination was unremarkable.

**Figure 1:**
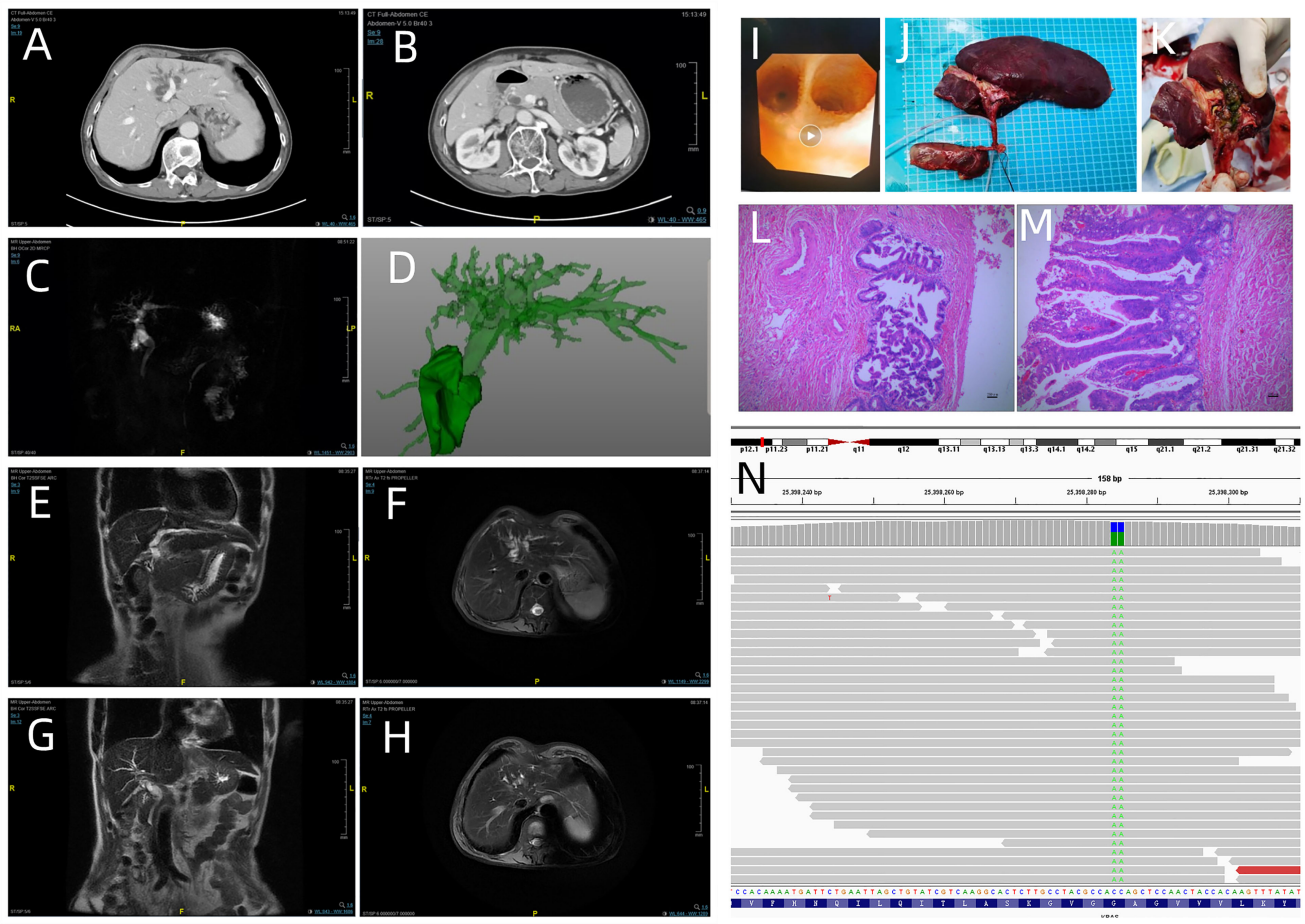
Enhanced computed tomography showed dilatation of the intrahepatic bile duct in the left lobe of the liver **(A)** and common bile duct **(B)**; **(C)** (MRCP) and **(D)** (CT three dimensional reconstruction) revealed the dilatation of extrahepatic and intrahepatic bile duct; **(E)** (coronal view) and **(F)** (axial view). Irregular dilatation and thickening of the bile duct are visible in the left hepatic lobe; **(G)** (coronal view) and **(H)** (axial view) show dilatation of the bile duct in the right left hepatic lobe. **(I)** Intraoperative cholangioscopy findings: biliary papillomatosis in the left hepatic duct. **(J, K)** Macroscopic findings: the bile duct is filled with greenish- yellow mucus- like sludge. (**L**: magnification, x40 and **M**: magnification, x100; hematoxylin and eosin staining) Microscopic findings: the intrahepatic bile duct, common bile duct and cystic duct were lined by papillary growth neoplasia with high-grade intraepithelial neoplasia. **(N)** Next-generation sequencing results revealed the mutation of KRAS codon 12 (p.G12F).

Enhanced computed tomography (CT) at our hospital revealed interruption of the intrapancreatic common bile duct (**Figure 1B**) and dilatation of the extrahepatic and intrahepatic bile duct, which was especially obvious in the left lobe of the liver (**Figure 1A**). To evaluate dilatation of the bile duct, magnetic resonance cholangiopancreatography (MRCP) was performed. MRCP showed dilatation of the extrahepatic and intrahepatic bile ducts, irregular dilatation and thickening of the bile duct in the left lobe of the liver (**Figures 1C–H**). At the same time, there were localized nodular prominences in the bile duct lumen. Laboratory values on admission were as follows: tumor markers, alpha-fetoprotein (AFP) 2. 6 ng/mL (normal range 0.00-8.78 ng/mL), ferritin (Fer) 153.96 ng/mL (normal range 21.80–274. 66 ng/mL), carcinoembryonic antigen (CEA) 2. 57 ng/mL (normal range 0.00–5. 00 ng/mL), and cancer antigen 19-9 (CA199) 47. 85 U/mL (normal range 0.00–37.0 U/mL). A routine blood examination was not abnormal. The level of γ-glutamyltranspeptidase (γ-GTP) was elevated, 109 U/L (normal range 7–49 U/L) (**Table 1**).

**Table 1:** Laboratory data before and after the surgery.

	Case 1			Case 2			Normal range
	Pre-operation	Post-operation (6 days)	Post-operation (8 months)	Pre-operation	Post-operation (10 days)	Post-operation (6 months)	
Tumor markers							
AFP	2. 6	–	–	1.97	–	1.54	0.00-8.78 ng/mL
Fer	153.96	–	–	288.81	–	195.62	21.80–274. 66 ng/mL
CEA	2. 57	–	–	4.91	–	2.65	0.00–5. 00 ng/mL
CA199	47. 85	–	28.65	95.00	–	35.55	0.00–37.00 U/mL
Routine blood tests							
WBC	4.21	7.15	5.74	9.81	9.02	–	3.50-9. 50×10^9/L
RBC	4.69	3.84	4.46	5.74	4.22	–	4.30 -5.80×10^12/L
Hb	139	118	131	163	122	–	130-175g/L
PLT	216	170	149	171	139	–	125-350×10^9/L
NEU%	65.3	69.7	62.4	77.0	70.4	–	40.0-75.0%
Liver function tests							
ALB	45	37	44	41	34	–	35-55 g/L
ALT	17	57	17	20	78	–	5-40 U/L
AST	19	24	21	17	27	–	5-40 U/L
ALP	90	58	42	104	68	–	40-150 U/L
γ-GTP	143	92	35	196	84	–	7-49 U/L

After the relevant preoperative examination was completed, laparoscopic hepatic segmentectomy (S2, S3, S4), resection of the common bile duct, cholecystectomy, and hepaticojejunostomy were performed. On intraoperative cholangioscopy, we found the left hepatic duct filled with mucus and papillary protrusions adhering to the surface of the left hepatic duct (**Figure 1I**). No abnormalities were detected in the common bile duct. Laparoscopic hepatic segmentectomy (S2, S3, S4), resection of the common bile duct, cholecystectomy, and hepaticojejunostomy were performed. The bile duct was filled with greenish -yellow mucus -like sludge (**Figures 1J, K**). Microscopically, the intrahepatic bile duct, common bile duct and cystic duct were lined by papillary growth neoplasia with high-grade intraepithelial neoplasia (**Figures 1L, M**). Gene detection was performed using DNA extracted from paraffin-embedded bile ducts. Gene detection revealed KRAS codon 12 (p. G12F) mutation, and the mutation frequency was 62.9% (**Figure 1N**).

The postoperative course was uneventful, and the patient was discharged from the hospital on the 7th postoperative day. The patient had no recurrence and no complications for 8 months after surgery.

### Case 2

A 67-year-old male with diabetes mellitus was admitted to our hospital due to finding dilatation of extrahepatic and intrahepatic bile ducts by accident during a routine medical examination 15 days ago. The patient was asymptomatic. He did not feel abdominal pain or abdominal distension. The patient did not have a history of viral hepatitis or alcoholic liver disease. There were no remarkable findings during the physical examination.

**Figure 2:**
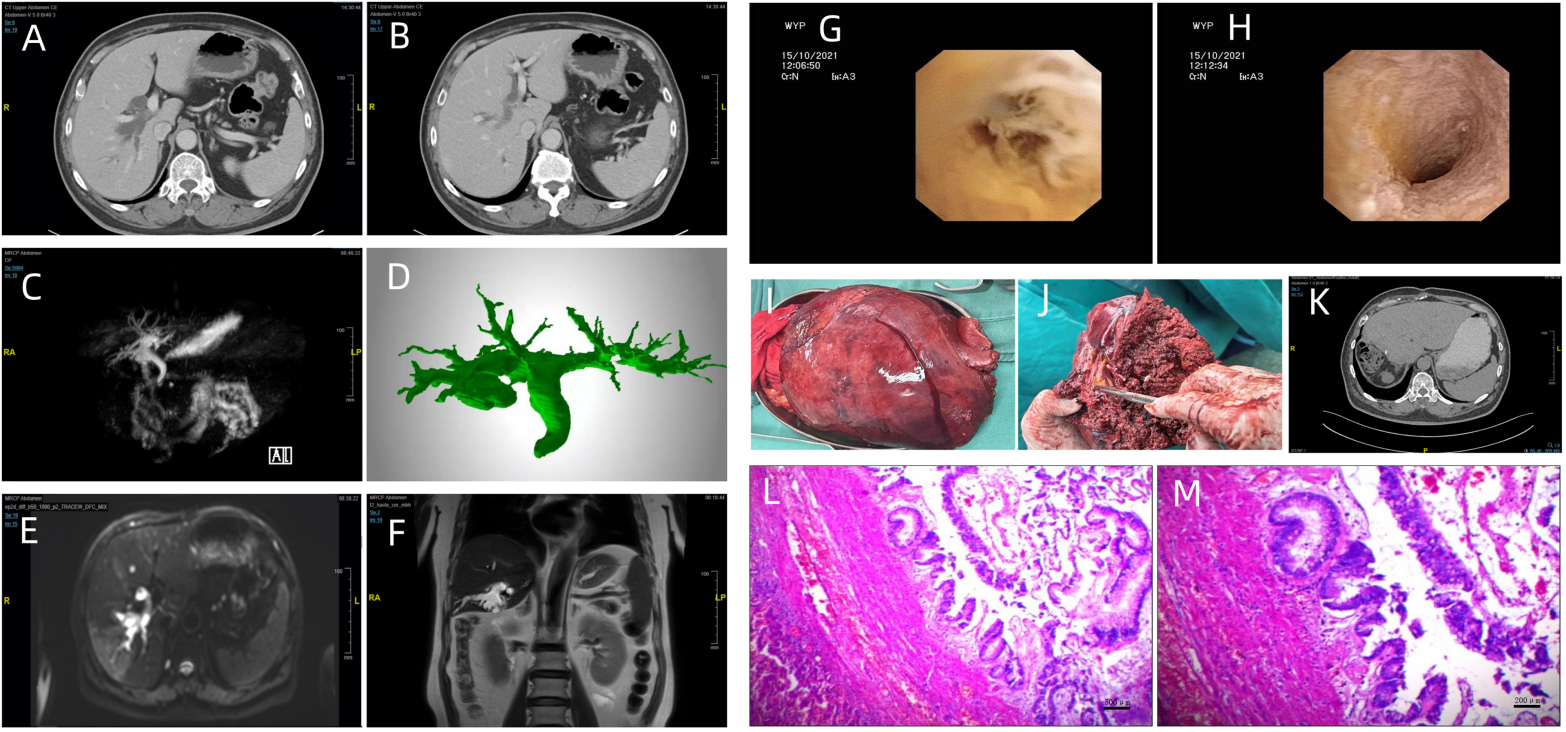
**(A, B)** Enhanced computed tomography demonstrated thickening of the distal common bile duct wall, luminal stenosis, dilatation of extrahepatic and intrahepatic bile duct, especially more evident in the right lobe of liver; **(C)** (MRCP) and **(D)** (CT three dimensional reconstruction) revealed the dilatation of extrahepatic and intrahepatic bile duct; **(E)** (diffusion-weighted image) The bile duct showed high intensity; **(F)** (coronal view) showed the dilatation of intrahepatic bile duct in the right lobe of liver; Intraoperative cholangioscopy revealed the common bile duct wall was full of the white flocculus **(G)** and rough **(H)**; Grossly, the lumen of intrahepatic bile duct was full of yellow, gelatinous, sticky mucous masses **(I, J)**; abdominal computed tomography (CT) showed homogeneous liver parenchyma without bile duct dilation **(K)**; Microscopically, Intraductal papillary neoplasm of the bile duct(IPNB)with low-grade intraepithelial neoplasia, focal high-grade intraepithelial neoplasia; A large amount of lymphocytes aggregated portal area with extensive bile duct proliferation (**L**: magnification, x40 and **M**: magnification, x100; hematoxylin and eosin staining).

Dynamic upper abdominal computed tomography (CT) showed thickening of the distal common bile duct wall, luminal stenosis and dilatation of extrahepatic and intrahepatic bile duct, which was especially pronounced in the right lobe of the liver with a diameter of approximately 15 mm at its widest point (**Figures 2A, B**). Magnetic resonance cholangiopancreatography (MRCP) revealed that the common hepatic duct and intrahepatic bile duct were dilated (**Figures 2C, D, F**). The bile duct wall showed high intensity on diffusion-weighted imaging (**Figures 2E**). Laboratory test results included tumor markers, alpha-fetoprotein (AFP) 1. 97 ng/mL (normal range 0.00-8.78 ng/mL), ferritin (Fer) 288.81 ng/mL (normal range 21.80–274. 66 ng/mL), carcinoembryonic antigen (CEA) 4. 91 ng/mL (normal range 0.00–5. 00 ng/mL), and cancer antigen 19-9 (CA199) 95.00 U/mL (normal range 0.00–37.0 U/mL). Routine blood tests revealed a white blood cell count of 9.81×109/L (normal range 3.50-9. 50×109/L) and a neutrophilic granulocyte percentage of 77.0% (normal range 40.0-75.0%). The level of γ-glutamyltranspeptidase (γ-GTP) was elevated, 196 U/L (normal range 7–49 U/L) (**Table 1**). The diagnosis of intraductal papillary neoplasm of the bile duct (IPNB) was suspected based on the imaging and laboratory findings.

Intraoperative cholangioscopy was performed to evaluate the common bile duct. The common bile duct wall was rough and full of white flocculus (**Figures 2G, H**). Therefore, the patient underwent robot-assisted laparoscopic hepatic segmentectomy (S5, S6, S7, S8), resection of the common bile duct, hepaticojejunostomy and cholecystectomy. Grossly, the intrahepatic bile duct lumen was filled with yellow, gelatinous, sticky mucous masses. The diameter of the largest one was 18 mm. (**Figures 2I, J**) Pathologic findings were as follows: Intraductal papillary neoplasm of the bile duct (IPNB) with low-grade intraepithelial neoplasia, focal high-grade intraepithelial neoplasia; a large number of lymphocytes aggregated in the portal area with extensive bile duct proliferation (**Figures 2L, M**). The patient refused gene detection for financial reasons.

The patient’s postoperative course was uneventful. The patient was discharged from our department 13 days after surgery. The patient was followed up for 6 months after the operation, and there were no signs of recurrence. (**Figure 2K**).

## Discussion

Intraductal papillary neoplasm of the bile duct (IPNB) is a rare bile duct tumor characterized by intraductal papillary or villous neoplasms covered by neoplastic epithelium with fine fibrovascular stalks in the dilated bile ducts (1).

IPNB is mainly found in patients in Eastern countries, such as Japan, and Korea, where hepatolithiasis and clonorchiasis, which are known to be major risk factors for IPNB, are endemic (4). IPNB shows a slight male predominance, and most patients are between 50 and 70 years of age is reported (4, 5).

However, the pathogenesis and nature of IPNB are still unclear. It is likely caused by cholestasis, biliary tract infection, and biliary tract cancer. Studies have found a mechanism for biliary tract cancer due to the progression of chronic inflammation to multistage carcinogenesis and eventually hyperplasia-dysplasia-carcinoma (6). Furthermore, chronic inflammation induces the production of reactive oxygen species and reactive nitrogen species, resulting in DNA damage, which plays an important role in carcinogenesis (7). IPNB symptoms include recurrent and intermittent abdominal pain, cholangitis, and jaundice. However, some patients are asymptomatic (3, 8). Histological types of IPNB have been classified into the following four types: gastric, intestinal, pancreaticobiliary, oncocytic, and IPMN, depending on morphologic appearance and mucin staining properties (9). GNAS and KRAS mutations detected in 50% and 46.2% of IPNBs are common in IPNBs. KRAS plays an important role in regulating cell growth and differentiation (10), A recent study has shown that KRAS mutation was detected in one third of intrahepatic cholangiocarcinoma (ICC) and also one third of biliary intraepithelialneoplasia (BilINs) associated with hepatolithiasis (11). Moreover, the KRAS mutation rate in high-grade IPNBs, and invasive IPNBs is significantly higher than low- and intermediate-grade IPNBs. So KRAS mutation may contribute to the pathogenesis of IPNBs (12, 13). Research shows that the 5-year survival rate of patients undergoing R0 resection is 59.7%. However, it is still lower than that for patients with benign diseases and better than intrahepatic cholangiocarcinoma (14, 15).

Surgical resection is the first line treatment for patients with IPNB without distant metastasis because of recurrent cholangitis and obstructive jaundice (1, 16). Liver transplantation and pancreaticoduodenectomy can be theoretically regarded as the only curative treatment. However, liver transplantation is unsuitable for patients with advanced tumor invasion or positive lymph nodes (4). Recently, new approaches, such as RFA and APC, have been helpful for patients who are not candidates for surgery due to age or physical condition (17, 18).

To further investigate the clinical features and prognosis of IPNB, we gathered case reports of IPNB in PubMed (17–55). We searched PubMed for “Intraductal papillary neoplasm of the bile duct” and “case report” during 2002 to 2021. Case reports were included if they reported any aspect of the clinicopathological or surgical characteristics of patients with IPNB confirmed by pathology. Most patients were more than 45 years of age, with a median age of 69 years. Only two patients were younger than 45 years of age. Males slightly predominated the case reports. The geographic distribution of patients was not different from other reviews, with IPNB mainly in Asia. There was only one patient found in Africa. However, the patients was Japanese. The tumor size ranged from 10.6-130. 0 mm, with a median size of 52. 9 mm. IPNB can develop anywhere along the biliary tree, with 25.0% in the right lobe of the liver, 32.5% in the left lobe of the liver, 12.5% in the CBD, 17.5% in the extrahepatic duct and liver and 12.5% in other locations, including the whole liver or gallbladder. The common radiologic findings for IPNB are bile duct dilatation, intraductal mass and cystic lesion. Among all cases, 28 included information on tumor markers, the CA 19-9 of 10 cases was elevated, and 18 were not elevated. The type of surgery depended on various anatomical locations of the tumor and the physical qualifications of the patients. Hepatectomy, which accounted for 40%, was the main surgical approach. Twenty-five percent of patients underwent hepatectomy, extrahepatic bile duct resection, and hepaticojejunostomy. Five percent of patients underwent hepatectomy and segmental pancreatectomy. A total of 7.5% of patients underwent pancreatoduodenectomy. As a palliative treatment, endoscopic treatment is necessary for patients with poor physical conditions. It is worth mentioning that RFA and APC accounted for 7.5% of promising therapeutic strategies and are expected to bring new approaches for IPNB (**Table 2**). IPNB lesions frequently contain invasive components. Twenty-three cases (57.5%) contained evidence of high- grade cholangiocarcinoma, while 17 cases (42.5%) ranged from no invasion to medium-grade cholangiocarcinoma. Immunohistochemical data were available for 26 cases, from which we found that CK7 (42.3%), MUC5AC (53.8%) and MUC6 (53.8%) expression was common in the IPNB. Based on the recurrence-free survival (RFS) reported in 23 cases, we found that the range of RFS was 4.0-39.0 months, with a median of 14.0 months (**Table 3**).

**Table 2:** The demographic characteristics, clinical features and surgical approaches of IPNB.

Feature	N(%)
Age (years)	
33-45	2 (5.0%)
45-57	4 (10.0%)
57-69	15 (37.5%)
69-81	12 (30.0%)
81-93	7 (17.5%)
Sex	
Male	22 (55.0%)
Female	18 (45.0%)
Region Asia America Europe Africa	28 (70.0%) 4 (10.0%) 7 (17.5%) 1 (2.5%)
Tumor size (mm)	Range (10.6-130.0) Median (52.9)
Tumor location	
The right lobe of the liver	10 (25.0%)
The left lobe of the liver	13 (32.5%)
CBD	5 (12.5%)
Extrahepatic duct and liver	7 (17.5%)
Other	5 (12.5%)
Imaging findings	
Solid mass	9 (22.5%)
Cystic lesion	6 (15.0%)
Dilation of the bile duct	7 (17.5%)
Solid mass;cystic lesion	5 (12.5%)
Solid mass;dilation of the bile duct	5 (12.5%)
Cystic lesion;dilation of the bile duct	4 (10.0%)
Solid mass;cystic lesion;dilation of the bile duct	3 (7.5%)
Unknown	1 (2.5%)
Tumor marker (CA 19-9)	
Elevated	10 (25.0%)
Not elevated	18 (45.0%)
Unknown	12 (30.0%)
Surgical approaches	
Hepatectomy	16 (40.0%)
Hepatectomy;extrahepatic bile duct resection;hepaticojejunostomy	10 (25.0%)
Hepatectomy;segmental pancreatectomy	2 (5.0%)
Pancreatoduodenectomy	3 (7.5%)
Endoscopic treatment	3 (7.5%)
RFA and APC	3 (7.5%)
Other	2 (5.0%)
Unknown	1 (2.5%)

**Table 3:** The pathological features and RFS of IPNB.

Tumor grade	N(%)
no invasion	5/40 (12.5%)
low grade	5/40 (12.5%)
low to intermediate grade	3/40 (7.5%)
intermediate grade	2/40 (5.0%)
intermediate to high grade	2/40 (5.0%)
high grade	8/40 (20%)
I PNB with cholangiocarcinoma	15/40 (37.5%)
Immunohistochemistry (+)	
CK7	11/26 (42.3%)
MUC5AC	14/26 (53.8%)
MUC6	14/26 (53.8%)
MUC1	5/26 (19.2%)
MUC2	3/26 (11.5%)
RFS (month)	Range (4.0-39.0) Median (14.0) N(23)

On further review of the literature, we could conclude that IPNB mainly behaves as in imaging solid mass, cystic lesion and dilation of the bile duct in imaging. Surgical resection is the major treatment for patients in fine condition, and interventional therapies such as RFA and APC are good choices for palliative treatments. Although most IPNB cases are high-grade intraepithelial neoplasia or invasive carcinoma in microscopically, surgical resection could make patients get satisfactory prognosis.

## Conclusion

In summary, we report two cases of IPNB, a rare tumor of the hepatobiliary system, and analyze published case reports about IPNB. Since IPNB has a high potential for transforming into an invasive lesion, R0 surgical resection is preferred. At the same time, RFA and APC may be new palliative approaches for treating IPNB.

## Data Availability Statement

The datasets presented in this study can be found in online repositories. The names of the repository/repositories and accession number(s) can be found in the article/supplementary material.

## Author Contributions

BL designed the case report. ZL, QL, and WT participated in the operation and management of the patients. BL, ZM, and ML prepared radiological and histology figures. BL, ZL reviewed the literature and drafted the article. All authors contributed to the article and approved the submitted version.

## Conflict of Interest

The authors declare that the research was conducted in the absence of any commercial or financial relationships that could be construed as a potential conflict of interest.

## Publisher’s Note

All claims expressed in this article are solely those of the authors and do not necessarily represent those of their affiliated organizations, or those of the publisher, the editors and the reviewers. Any product that may be evaluated in this article, or claim that may be made by its manufacturer, is not guaranteed or endorsed by the publisher.
